# P-selectin blockade ameliorates lupus nephritis in MRL/lpr mice through improving renal hypoxia and evaluation using BOLD-MRI

**DOI:** 10.1186/s12967-020-02284-1

**Published:** 2020-03-05

**Authors:** Liwen Zhang, Sheng Chen, Yan Liu, Xueqin Xu, Qianying Zhang, Shuxin Shao, Weiming Wang, Xiao Li

**Affiliations:** 1 Department of Nephrology, Ruijin Hospital, Shanghai Jiao Tong University School of Medicine, 197 Ruijin Er Road, Shanghai, 200025 People’s Republic of China; 2 Department of General Surgery, Ruijin Hospital, Shanghai Jiao Tong University School of Medicine, Shanghai, 200025 People’s Republic of China; 3 Department of Radiology, Ruijin Hospital, Shanghai Jiao Tong University School of Medicine, Shanghai, 200025 People’s Republic of China

**Keywords:** Lupus nephritis, Hypoxic, Tubulointerstitial fibrosis, BOLD-MRI

## Abstract

**Background:**

Lupus nephritis is one of the most common and severe complications of systemic lupus erythematosus, of which poor prognosis is indicated by aggravated renal hypoxia and tubulointerstitial fibrosis. Cell adhesion molecules play a key role in the progression of lupus nephritis tubulointerstitial lesion, including P-selectin, which mediates the rolling of leukocytes and subsequent adhesion and infiltration and then initiates the inflammatory immune response and ischemia and hypoxia injury. However, the effects and mechanisms of P-selectin in lupus nephritis remain to be investigated, and a noninvasive measurement of lupus nephritis tubulointerstitial hypoxia and fibrosis remains to be explored.

**Methods:**

Thirty-four MRL/lpr mice were randomly divided into the following three groups: MRL/lpr, saline, and anti-P-selectin, which consisted of no treatment, treatment with normal saline, and treatment with anti-P-selectin monoclonal antibody (mAb) from 12 to 16 weeks of age, respectively. Ten male C57BL/6 mice of the same age served as normal controls. 24-h urinary protein, urinary albumin–creatinine ratio, and periodic acid–Schiff were used to assess kidney damage; Western blot or immunohistochemical staining of the hypoxia probe Hypoxyprobe™-1, hypoxia-inducible factor 1α (HIF-1α), and CD31 were used to evaluate hypoxia in renal tissue; and NADPH oxidase subunit gp91phox and p22phox were used to examine renal oxidative stress. The correlation between kidney injury and blood oxygen level–dependent magnetic resonance imaging (BOLD-MRI) was calculated to assess the clinical value of BOLD-MRI.

**Results:**

P-selectin is upregulated in lupus nephritis. Blocking P-selectin with mAb alleviated renal tubulointerstitial fibrosis, renal hypoxia, and peritubular capillary loss, without alteration of the levels of lupus activity indicators, anti-dsDNA antibody, or complement C3. BOLD-MRI showed that the reduced R2* values in the renal cortex and medulla of lupus mice were increased when treated with anti-P-selectin mAb as compared with those treated with normal saline, which were negatively correlated with Hypoxyprobe™-1 hypoxia probe and the expression of HIF-1α.

**Conclusions:**

Early intervention of lupus nephritis with anti-P-selectin mAb can significantly improve the hypoxic state of the kidney and reduce the severity of tubulointerstitial lesions. BOLD-MRI techniques are noninvasive and can dynamically evaluate the changes in renal lesions and intrarenal oxygenation levels before and after treatment in lupus nephritis.

## Background

Systemic lupus erythematosus (SLE) is a systemic autoimmune disease affecting various organs. Lupus nephritis (LN) is one of the most common and severe complications of SLE, and some cases eventually progress to end-stage renal disease (ESRD). It is reported that renal hypoxia and tubulointerstitial fibrosis are associated with the poor prognosis in LN [1], and cell adhesion molecules play a key role in the progression of LN tubulointerstitial lesion [2, 3]. P-selectin, an adhesion molecule expressed on the surface of activated endothelia and platelets, mediates the rolling of leukocytes and subsequent adhesion and infiltration [4], which initiate the inflammatory immune response and ischemia and hypoxia injury [5, 6]. However, the effects and mechanisms of P-selectin in glomerulonephritis, especially LN, remain to be explored.

Inflammation caused by immune complex deposition in peritubular capillary (PTC) subendothelium, tubular basement membrane, or interstitium leads to tubulointerstitial lesion and LN progression, and tubulointerstitial hypoxia is a common mechanism for the development of various chronic kidney diseases into ESRD. At present, there is still a lack of ideal noninvasive methodology in the clinical evaluation of LN tubulointerstitial hypoxia or fibrosis. Renal diffusion-weighted imaging (DWI) combined with blood oxygenation level–dependent (BOLD) imaging has been used as a novel magnetic resonance (MR) functional examination technique that reflects changes in renal ischemia, hypoxia, and medullary oxygenation levels [7–9]. Pedersen and colleagues [10] confirmed that the apparent relaxation rate (R2*) value is strongly associated with regional renal oxygen content measured by oxygen-sensitive microelectrodes in pigs and have promoted BOLD magnetic resonance imaging (MRI) as a noninvasive estimate of renal partial oxygen pressure. In previous research, we assessed the utility of BOLD and DWI MRI in discrimination of renal involvement and pathological changes in 65 LN patients and 16 healthy volunteers and found a negative correlation between R2* values of the renal medulla and proteinuria (*r* = − 0.244, *P* < 0.05) as well as the degree of tubulointerstitial lesion (*r* = − 0.242, *P* < 0.05) [11]. However, investigations on the application value of BOLD-MRI in evaluating the damage of LN are still limited [12].

Here, we investigate the pathogenesis of LN tubulointerstitial lesion and demonstrated the renoprotective role of anti-P-selectin monoclonal antibody (mAb) in renal function, pathological characteristics, and tubulointerstitial hypoxia status of an LN animal model (MRL/lpr mice). Our findings suggest that blocking P-selectin with mAb alleviates renal tubulointerstitial fibrosis, renal hypoxia, and PTC loss without alteration of the levels of lupus activity indicators, anti-dsDNA antibody, or complement C3. BOLD-MRI showed that the R2* of the renal cortex and medulla of lupus mice treated with anti-P-selectin mAb was higher than that of the saline control littermates, promoting BOLD-MRI as a proper measurement of renal hypoxia in LN.

## Methods

### Animal experiments

Animal maintenance and experimental procedures were approved by the Animal Care Committee of Ruijin Hospital, Shanghai Jiao Tong University School of Medicine (Shanghai, China). Thirty-four MRL/lpr mice aged 11 weeks were introduced and cultivated by the Shanghai Experimental Animal Center of the Chinese Academy of Sciences from the Jackson Laboratory (USA), and 10 male C57BL/6 mice of the same age served as normal controls. For studies involving the role of anti-P-selectin mAb in LN, MRL/lpr mice were randomly divided into the following three groups: MRL/lpr, saline, and anti-P-selectin groups, which consisted of no treatment, treatment with normal saline, and treatment with anti-P-selectin mAb (clone RB40.34, IgG1λ; BD Biosciences, San Diego, California, United States) intraperitoneally at 2 mg/kg body weight twice a week from 12 to 16 weeks of age, respectively. Mice were housed in a specific pathogen-free room at a constant temperature of 22 ± 2 ℃ and a constant humidity of 50 ± 5% under a 12-h day/night cycle. Mice were given free access to chow and water.

### Physiologic parameters

Before the mice were euthanized, they were provided water ad libitum, and 24-h urine was collected in metabolic cages. The urinary albumin concentration was measured using a mouse albumin enzyme-linked immunosorbent assay (ELISA) quantitation set (Bethyl Laboratories, Inc., Montgomery, TX, USA). The urinary creatinine concentration in the same sample was measured using the QuantiChrom™ Creatinine Assay Kit (BioAssay Systems, Hayward, CA, USA) according to the manufacturer’s protocol. The serum was collected through the post–glomus venous plexus after anesthetization; anti-dsDNA (FUJIFILM Wako Shibayagi Corporation, Japan) and complement C3 (Icllab, Portland, OR, USA) levels were measured with ELISA kits according to the manufacturer’s protocol.

### Kidney histopathology

The kidneys were removed from anesthetized mice and immediately fixed in 4% paraformaldehyde, embedded in paraffin, and sectioned at 4 μm. The sections were stained with periodic acid–Schiff (PAS). PAS micrographs were observed by two renal pathologists independently to estimate semi-quantitative scores for glomerular and tubulointerstitial areas according to the area affected by the lesion: 0 (no), 1 (≤ 25%), 2 (25% to 50%), 3 (≥ 50%), in which glomerular 0–9 points, including mesangial cell proliferation 0–3 points; mesangial matrix hyperplasia 0–3 points; sclerosis 0–3 points; tubulointerstitial 0–9 points, including renal tubular atrophy 0–3 points; interstitial fibrosis 0–3 points; interstitial cells infiltration 0–3 points.

### Immunohistochemistry staining

One hour before the sacrifice of mice, the hypoxia probe Hypoxyprobe™-1 (Hypoxyprobe, Inc., Burlington, MA, USA) at 60 mg/kg body weight was injected intraperitoneally, and the intensity and distribution area were analyzed by immunohistochemical (IHC) staining according to the manufacturer’s protocol. IHC staining was performed on paraffin-embedded kidney sections following standard procedures by incubating the sections in a primary antibody against P-selectin (Cell Signaling Technology, Inc., Danvers, MA, USA), hypoxia-inducible factor 1α (HIF-1α; Novus Biologicals, Inc., Littleton, CO, USA), and CD31 (Abcam, Cambridge, MA, USA) at 4 ℃ overnight. After washing, the sections were incubated with biotinylated secondary antibodies, followed by incubation with an avidin–biotin–peroxidase complex for DAB substrate development using the ABC kit (Vector Laboratories, Burlingame, CA, USA) at room temperature, and they were mounted using Aqua PolyMount (Polysciences, Inc., Warrington, PA, USA). Images were acquired using a Leica DM1000 microscope with a digital camera. The results of immunohistochemistry were determined by two renal pathologists independently according to the distribution of light staining in renal tissue with a semi-quantitative score of 0–3, in which 0 indicates negative staining; 1 point, occasional positive staining; 2 points, focal positive staining; and 3 points, diffuse positive staining.

### Western blot analysis

Renal cortical tissues were ground and lysed, and cells were collected and lysed in RIPA buffer containing protease inhibitor cocktail. Equal amounts of protein samples were loaded on sodium dodecyl sulfate polyacrylamide gels, transferred to polyvinylidene difluoride membranes (Millipore, MA, USA), probed with antibodies, and visualized with the Luminescent Imaging Workstation (Tanon, China). The band intensities were quantified using ImageJ. Mouse anti–β-actin antibody (Sigma, MA, USA) was used as loading control.

### Renal BOLD-MRI examination

Mice were fasted for 4 to 8 h before BOLD-MRI. After anesthetization, mice were placed in a prone position. The limbs were fixed, and the animal-specific eight-channel high-resolution head coil was placed. The T1WI, T2WI, DWI, and BOLD images of kidney cross section were scanned using a GE Signa Excite 3.0T MR scanner. For cross-sectional T1WI and T2WI imaging, sequences included fast spin echo (FSE) T1WI [repetition time (TR)/echo time (TE) 540/12.7 ms], FSE T2WI (TR/TE 4000/46.2 ms), layer thickness 2.4 mm, layer spacing 0.2 mm, field of view (FOV) 6 cm × 6 cm, and matrix 256 × 224. The scope of the scan included both kidneys. For cross-sectional BOLD imaging, sequences included multiecho fast gradient-echo sequence, 8 gradient echoes, TR 110 ms, TE 2.0–27.5 ms, layer thickness 2 mm, and spacing 0.2 mm, with 5 levels of coronal imaging centered on the renal hilum. Parameters were set as flip angle 20°, bandwidth 125, number of excitations (NEX) 1, FOV 15 cm × 15 cm, and matrix 224 × 192 (8 frames per layer). The data were transferred to the ADW4.2 workstation, and the R2* values of the renal cortex and medulla were measured by Functool software on the image near the renal hilum. The R2* value measurement method consisted of the following: the window width/window position of the R2* image was adjusted to about 60.0/5.0, and the R2* color map of the kidney was used to measure the R2* value of the renal cortex. The region of interest (ROI) was posited on the clear boundary of the cortex and medulla manually, and 3 ROIs were placed on each side of the kidney cortex and medulla to calculate their average [11].

### Statistical analyses

The group data were expressed as the mean ± standard error of the mean (SEM). Comparisons between the two groups were performed using an unpaired *t* test after determining the distribution and variance of the data. One-way analysis of variance (ANOVA) followed by Tukey’s multiple comparisons test was used when more than two groups were present. All tests were two-tailed, and *P* < 0.05 was considered to be a statistically significant result.

## Results

### P-selectin expression in the kidney of MRL/lpr lupus mice

**Fig. 1:**
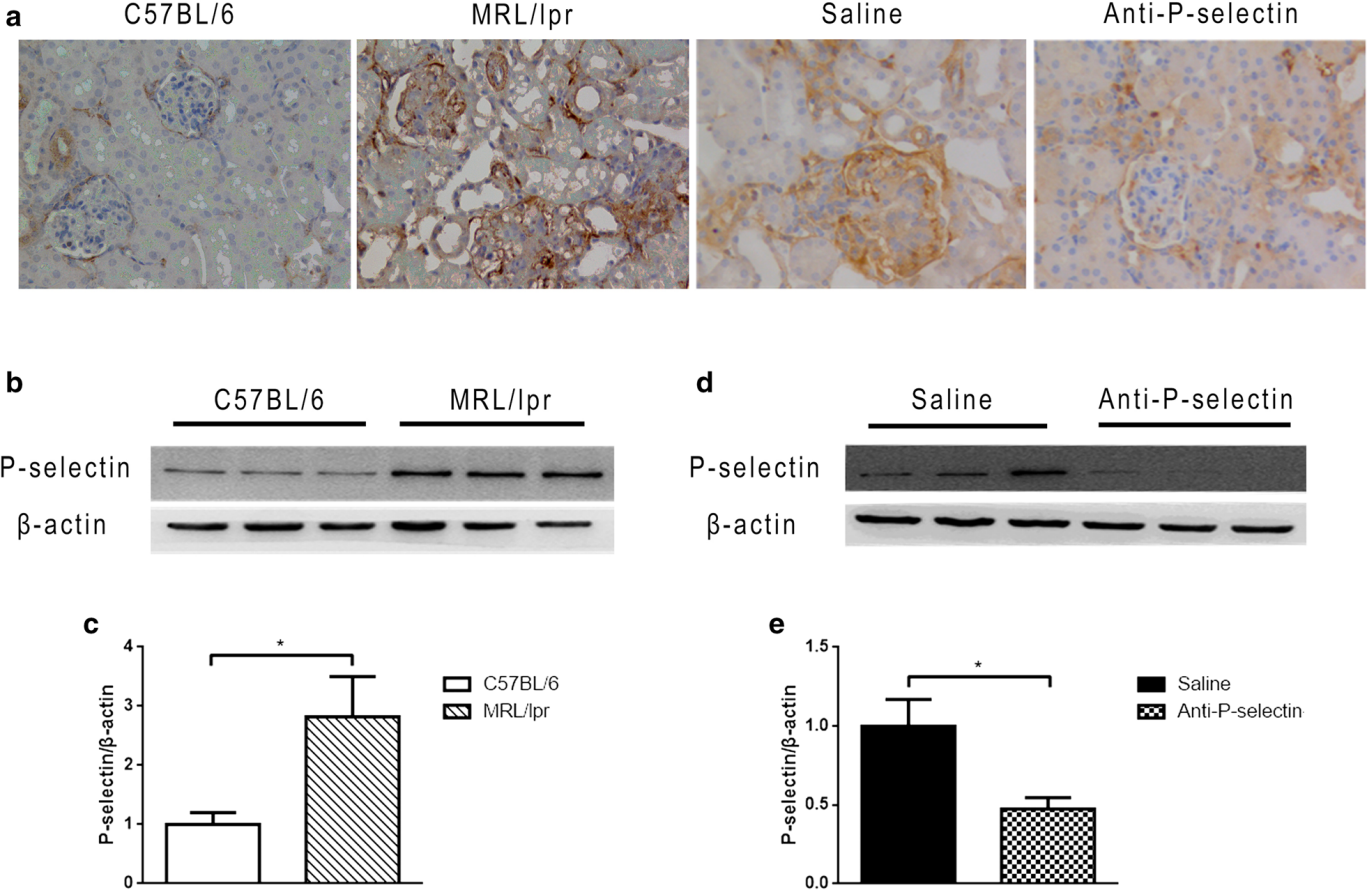
Anti-P-selectin mAb treatment neutralized the increased P-selectin expression in lupus nephritis. **a** Representative immunostaining micrographs of P-selectin show increased P-selectin expression in the glomeruli and tubulointerstitium of MRL/lpr mice and the neutralization of P-selectin with anti-P-selectin mAb (magnification of ×400). **b** Western blot analysis was used to assess altered P-selectin expression in renal tissues from MRL/lpr mice. β-actin was used as a loading control. **c** The quantification of the average band intensity for P-selectin in **b**. The values of expression in the control group were set as 1. **d** The expression of P-selectin in the renal tissues of saline or anti-P-selectin mAb–treated MRL/lpr mice (the saline and anti-P-selectin groups) was determined by western blot analysis. **e** Densitometric analyses of the western blots in (D). The relative intensities of the bands were normalized to the intensities of the respective β-actin signal, and the value of the saline group was set as 1. The results are presented as the mean ± SD. *P < 0.05 between the two indicated groups, as analyzed by Student’s *t* test (n = 6–9 for each group)

We analyzed the expression level and distribution characteristics of P-selectin in renal tissues of MRL/lpr mice by IHC staining. The renal tissue of C57BL/6 mice in the control group expressed P-selectin at a low level, and the positive area of lupus mouse kidney increased as compared with that in the control group, moderately in glomeruli and distinctively in tubulointestitium (Fig. 1a). There was no change in the expression of P-selectin in MRL/lpr renal tissue after treatment with normal saline, but anti-P-selectin mAb blocked P-selection expression distinctively (Fig. 1a). Moreover, the expression changes of P-selectin in IHC images in Fig. 1a were confirmed by western blot. It was shown through western blot analysis that renal P-selectin expression was significantly upregulated in MRL/lpr mice compared with that in C57BL/6 mice (Fig. 1b, c), and the expression level of P-selectin in the anti-P-selectin group was significantly decreased compared with that in the normal saline group (Fig. 1d, e).

### Anti-P-selectin mAb ameliorated kidney injury in MRL/lpr mice

**Fig. 2:**
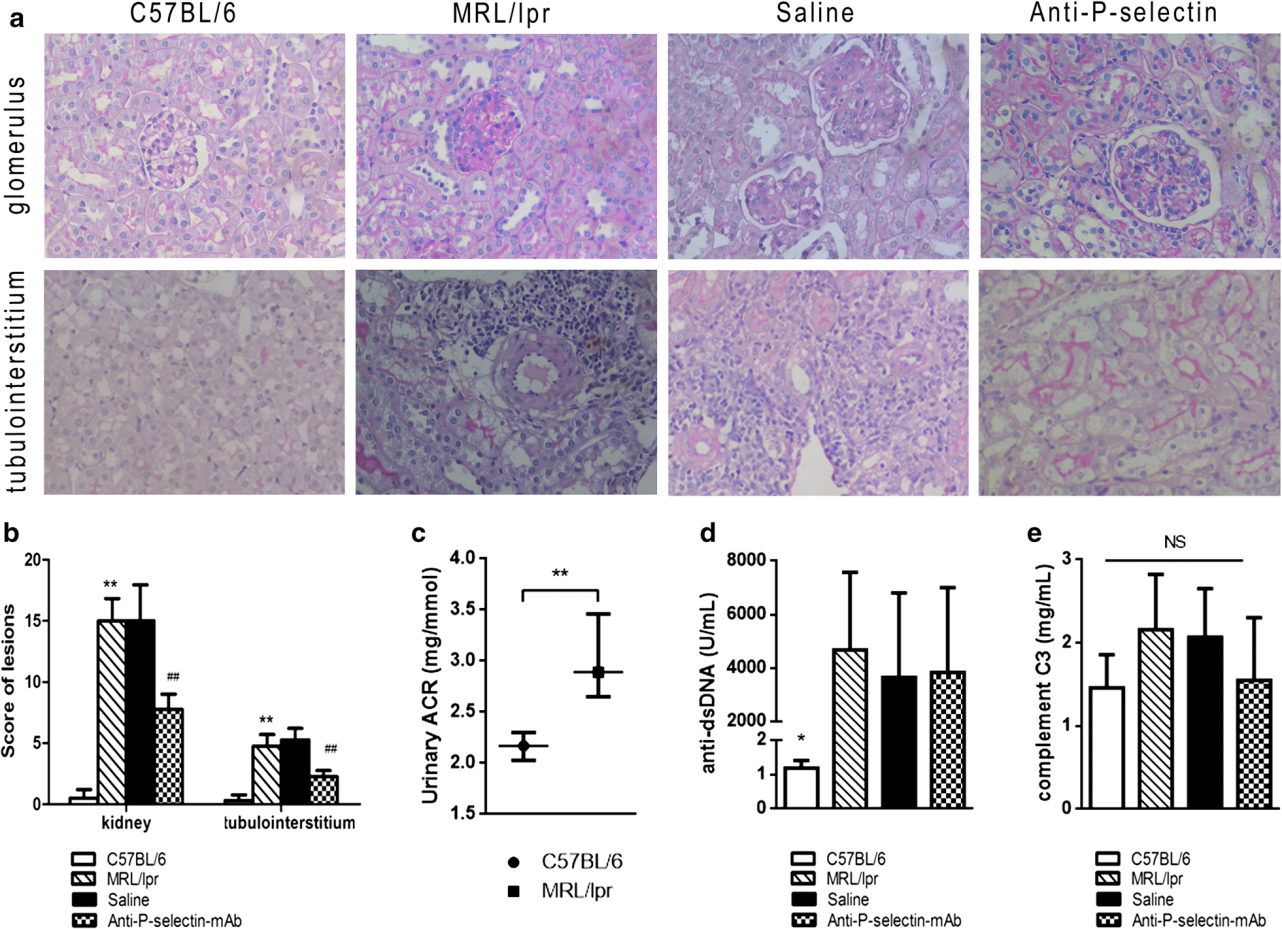
Anti-P-selectin mAb treatment ameliorated kidney injury in MRL/lpr mice. **a** Representative PAS staining micrographs of the glomerulus and tubulointerstitium in the C57BL/6, MRL/lpr, saline, and anti-P-selectin groups (magnification of ×400). **b** Semi-quantitative score of lesions in terms of kidney and tubulointerstitium. **P < 0.01 compared with C57BL/6 group; ^##^*P* < 0.01 compared with saline group. **c** Urinary albumin-creatinine ratio of the MRL/lpr group compared with the C57BL/6 group at the age of 12 weeks. The results are presented as the medium (first quartile, third quartile). **P < 0.01 between the indicated two groups (n = 10–13 for each group). **d** Serum anti-dsDNA levels of each group. *P < 0.05 compared with the MRL/lpr, saline, and anti-P-selection groups as analyzed by ANOVA followed by Tukey’s multiple comparisons test. **e** Complement C3 levels of each group. No statistical significance among groups

To investigate the effect of P-selectin blockade on LN, we evaluated the variation in histopathology and urinary protein and in MRL/lpr mice treated with normal saline or anti-P-selectin mAb. PAS staining micrographs showed severe glomerular, interstitial, and arteriolar lesions in both LN and normal saline–treated mice, which was characterized by mesangial expansion, basement membrane thickening, tubular epithelial detachment, interstitial inflammatory infiltration, and especially the remarkable accumulation of perivascular inflammatory cells (Fig. 2a). P-selectin blockade in MRL/lpr mice prevented these histopathologic lesions to a great extent, which presented as moderate mesangial proliferation, inconspicuous tubule atrophy, and less inflammatory infiltration (Fig. 2a). Moreover, both kidney and tubulointerstitial injury scores in the MRL/lpr group increased significantly as compared with those in the C57BL/6 group, and they decreased significantly in the anti-P-selectin group as compared with those in the saline group (Fig. 2b), indicating that anti-P-selectin mAb have a significant protective effect on LN injury.

The urinary albumin/creatinine ratio (ACR) of MRL/lpr mice was significantly higher than that of C57BL/6 mice at 12 weeks of age (Fig. 2c). We then evaluated the urinary protein levels and variation in MRL/lpr mice treated with normal saline or anti-P-selectin mAb (Table 1). After 4 weeks of anti-P-selectin mAb treatment (A2 group), the urinary ACR declined significantly compared with that of 12-week-old MRL/lpr mice (*P = 0.048); however, the urinary ACR did not show a difference after treatment with normal saline for 4 weeks (*P *= 0.22; A1 group). In addition, at 16 weeks of age, the 24-h urine protein level in group A2 was significantly lower than that in group A1 (^#^*P *= 0.02). We further investigated the effect of P-selectin blockade on urinary protein at different phases of LN, including from 12 to 20 weeks of age (P-selectin blockade and normal saline treatment as the B2 and B1 groups, respectively) and 16 to 20 weeks of age (P-selectin blockade treatment as the C1 group). The ACR of the B2 group (MRL/lpr mice treated with anti-P-selectin mAb at 12–20 weeks of age) was moderately lower than that of the normal saline control B1 group without statistical significance. Similar to the results in the A groups, the 24-h urinary protein in the B2 group at the age of 16 weeks was significantly lower than that in the B1 group (^##^*P *= 0.019), but there was no significant difference between the two groups at 20 weeks. Moreover, the 24-h urinary protein and ACR at 20 weeks in the B2 group were significantly lower than that in the C2 group that was treated with anti-P-selectin mAb from 16 to 20 weeks (^▲^*P *< 0.05). Based on these results, we chose MRL/lpr mice aged 12 weeks with 4 weeks of P-selectin blockade for the subsequent research.

**Table 1:** Urinary protein alteration in MRL/lpr mice with normal saline or anti-P-selectin monoclonal antibody treatment

	24-h urinary protein (mg)			Urinary protein concentration (mg/mL)			Urinary ACR (mg/mmol)		
	12 wks	16 wks	20 wks	12 wks	16 wks	20 wks	12 wks	16 wks	20 wks
Norma saline treatment									
A1	3.24 ± 1.47	4.22 ± 1.71	–	1.60 ± 0.82	2.70 ± 1.38**	–	4.36 ± 3.19	5.78 ± 4.43	–
B1	3.0 ± 1.71	4.08 ± 1.83	4.2 ± 5.95	1.63 ± 1.02	2.50 ± 1.39	2.79 ± 1.89	3.39 (2.74,6.32)	3.34 (2.78, 6.74)	5.02 (2.8, 33.77)
Anti-P-selectin mAb treatment									
A2	1.83 ± 0.83	1.19 ± 0.58^**#**^	–	1.57 ± 0.60	2.56 ± 1.55	–	2.69 ± 0.45	1.14 ± 0.10*	–
B2	3.67 ± 2.22	1.69 ± 0.89^**##**^	2.14 ± 1.41^▲^	1.94 ± 0.82	2.16 ± 0.80	3.30 ± 1.06	2.36 (2.14,3.11)	2.68 (1.24,3.21)	2.35 (2.0, 8.65)^▲^
C2	–	3.75 ± 1.99	3.94 ± 3.17	–	2.80 ± 0.47	3.58 ± 0.74	–	2.35 (2.0, 8.65)	10.07 (2.54,12.59)

The results are presented as the mean ± SD or median (first quartile, third quartile). Saline groups:A1 and B1 groups were treated with normal saline from 12 to 16 weeks and from 12 to 20 weeks, respectively. Anti-P-selectin groups: A2, B2, and C2 groups were treated with anti-P-selectin mAb from 12 to 16 weeks, from 12 to 20 weeks, and from 16 to 20 weeks, respectively

Compared with the baseline (that in 12 weeks for the same group), *, **P < 0.05. Compared with the same age saline group, ^#^, ^##^*P* < 0.05. Compared with the C2 group, ^▲^*P* < 0.05

We also detected anti–double-stranded DNA (dsDNA) antibody and complement C3 levels in serum to investigate the system activity of SLE in mice. The level of anti-dsDNA antibody in LN mice increased sharply (by about 5000-fold) as compared with that in the C57BL/6 control mice, but there was no significant difference in the level of anti-dsDNA antibody between the saline and anti-P-selectin mAb–treated groups (Fig. 2d). Complement C3 levels among the four groups were moderately various (Fig. 2e), as well as between different weeks of age (data not provided). These results demonstrated that P-selectin blockade protected renal injury in MRL/lpr LN mice without affecting the systemic SLE activity.

### Anti-P-selectin mAb attenuated renal hypoxia in MRL/lpr mice

**Fig. 3:**
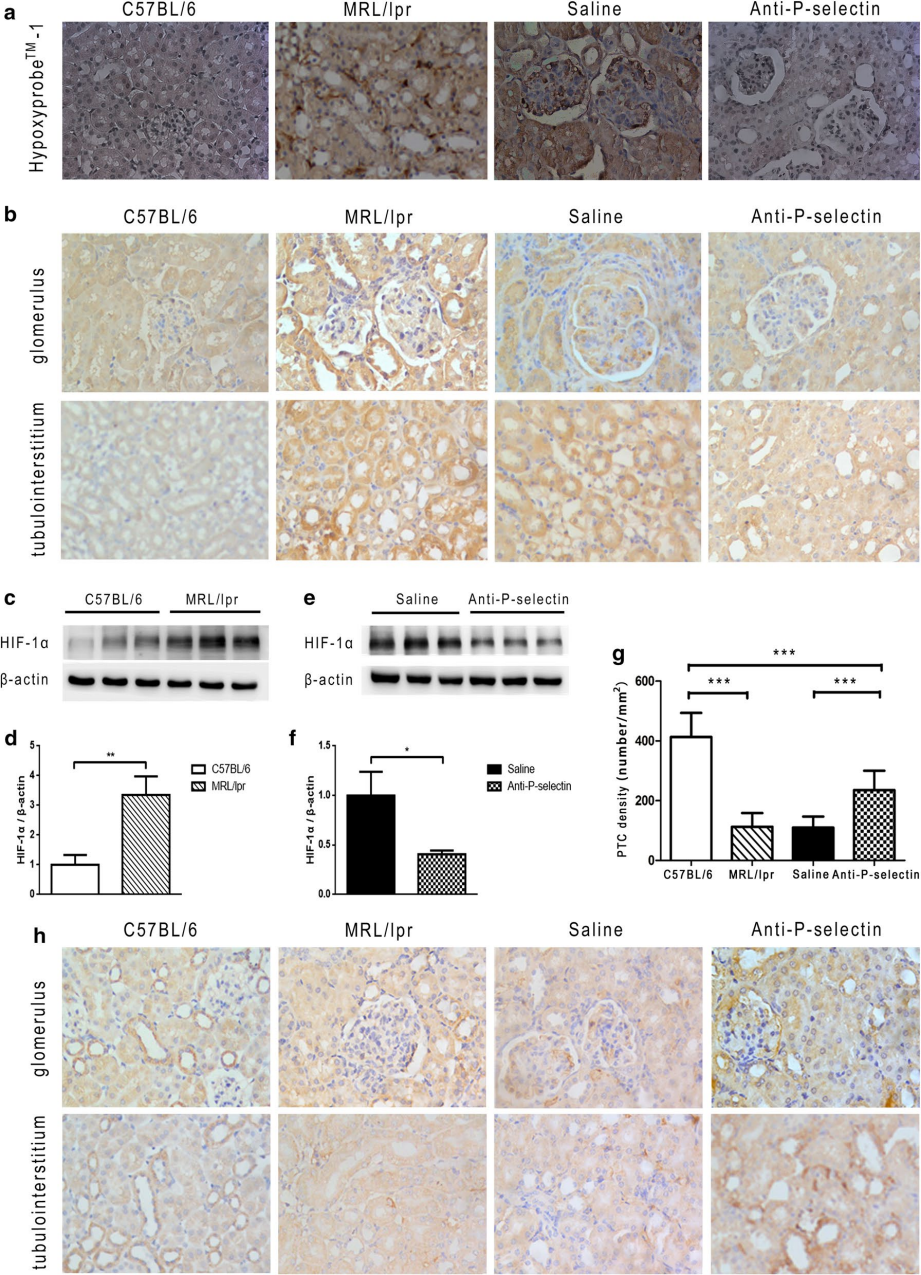
Anti-P-selectin mAb treatment ameliorated renal hypoxia in MRL/lpr mice. **a** Representative hypoxia probe in situ staining micrographs of by hypoxyprobe™-1 (magnification of ×400). **b** Immunohistochemistry staining of HIF-1α in the glomerulus and tubular interstitium (magnification of ×400). **c** Western blot analysis was used to assess altered HIF-1α expression in renal tissues from MRL/lpr mice. β-actin was used as a loading control. **d** The quantification of the average band intensity for HIF-1α in **c**. The values of expression in the control group were set as 1. **e** The expression of HIF-1α in the renal tissues of saline or anti-P-selectin mAb–treated MRL/lpr mice (the saline and anti-P-selectin groups) was determined by western blot analysis. **f** Densitometric analyses of the western blots in **e**. The relative intensities of the bands were normalized to the intensities of the respective β-actin signal, and the value of the saline group was set as 1. The results are presented as the mean ± SD. *P < 0.05 between the two indicated groups, as analyzed by Student’s *t* test (n = 6–9 for each group). **g** Peritubular capillary count (PTC) in the kidney of each group. ***P < 0.001 as analyzed by ANOVA followed by Tukey’s multiple comparisons test. **h** Immunohistochemistry staining of CD31 for PTC counting in the glomerulus and tubular interstitium (magnification of ×400)

We employed immunohistochemistry to detect the distribution of Hypoxyprobe™-1 hypoxic probe in renal tissue. It showed mild positive expression in the kidney of C57BL/6 mice; however, it was widely distributed in the renal tissue of MRL/lpr mice without intervention, including glomerular mesangial area, tubular epithelial cells, and interstitial and perivascular areas, suggesting severe hypoxia in MRL/lpr kidney. The positive area of Hypoxyprobe™-1 hypoxia probe in the kidney of the anti-P-selectin group declined as compared with that in the saline group (Fig. 3a). Moreover, HIF-1α was wildly expressed in the kidney, and the immunohistochemistry staining image in the C57BL/6 group showed moderately positive expression, but the positive area increased obviously in the MRL/lpr group and more distinctively in the interstitial and medulla area than in the glomeruli (Fig. 3b). There was no significant difference in HIF-1α expression between the saline group and the untreated MRL/lpr group, but that in the anti-P-selectin mAb intervention group was restored (Fig. 3b). Furthermore, we confirmed the variation in HIF-1α and the statistical significance among the groups in protein levels via western blot (Fig. 3c–f). Then, the distribution of capillary was determined with immunohistochemistry staining of CD31, an endothelial marker, and we found that the PTC count in the kidney of the MRL/lpr group reduced significantly as compared with that of the C57BL/6 group (Fig. 3g, h). After the intervention with anti-P-selectin mAb, the PTC count of renal tissue in lupus mice was significantly higher than that in lupus mice treated with normal saline (Fig. 3g, h).

**Fig. 4:**
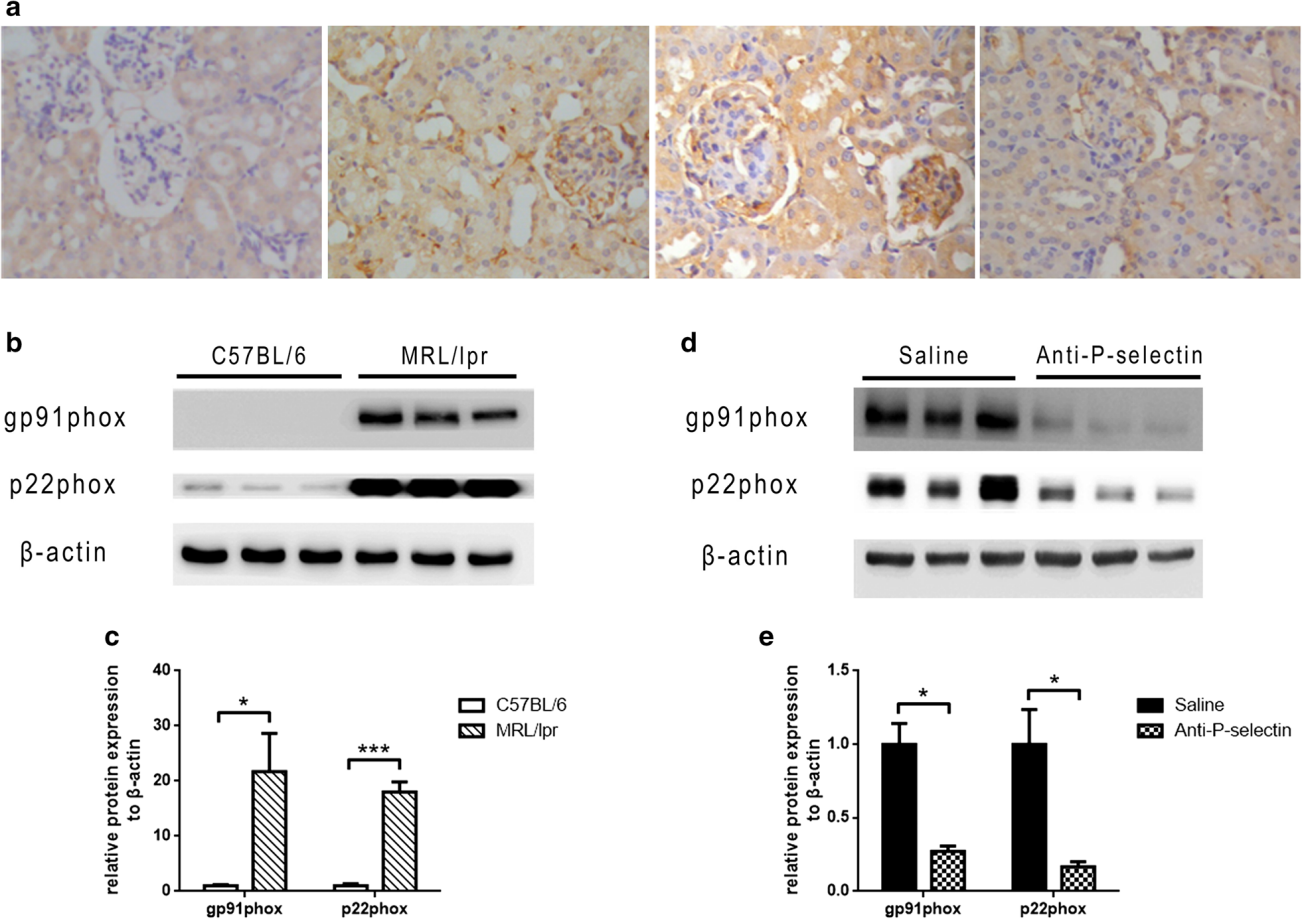
Anti-P-selectin mAb treatment ameliorated renal oxidative stress in MRL/lpr mice. **a** Representative immunohistochemistry staining micrographs of gp91phox in the C57BL/6, MRL/lpr, saline, and anti-P-selectin groups (magnification of ×400). **b** Western blot analysis was used to assess altered gp91phox and p22phox expression in renal tissues from MRL/lpr mice. β-actin was used as a loading control. **c** The quantification of the average band intensity for gp91phox and p22phox in **b**. The values of expression in the control group were set as 1. **d** The expression of gp91phox and p22phox in the renal tissues of saline or anti-P-selectin mAb–treated MRL/lpr mice (the saline and anti-P-selectin groups) was determined by western blot analysis. **e** Densitometric analyses of the western blots in **d**. The relative intensities of the bands were normalized to the intensities of the respective β-actin signal, and the value of the saline group was set as 1. The results are presented as the mean ± SD. *P < 0.05 between the two indicated groups, as analyzed by Student’s *t* test (n = 6–9 for each group)

In addition, the distribution of NADPH oxidase subunit gp91phox in mouse kidney was detected by immunohistochemistry to further assess renal oxidative stress. There was no obvious expression in C57BL/6 mouse kidney tissue (Fig. 4a, Line 1), while strong positive staining was detected in the mesangial and tubulointerstitial area of MRL/lpr mice (Fig. 4a, Line 2), and anti-P-selectin mAb treatment reversed the upregulation of gp91phox in MRL/lpr kidney (Fig. 4a, Line 3 and 4). The expression levels of NADPH oxidase subunits gp91phox and p22phox in MRL/lpr mice were significantly upregulated as evaluated by western blot (Fig. 4b, c), and they decreased after treatment with anti-P-selectin mAb (Fig. 4a, d, e).

### Evaluation of renal hypoxia in MRL/lpr mice through BOLD-MRI

**Fig. 5:**
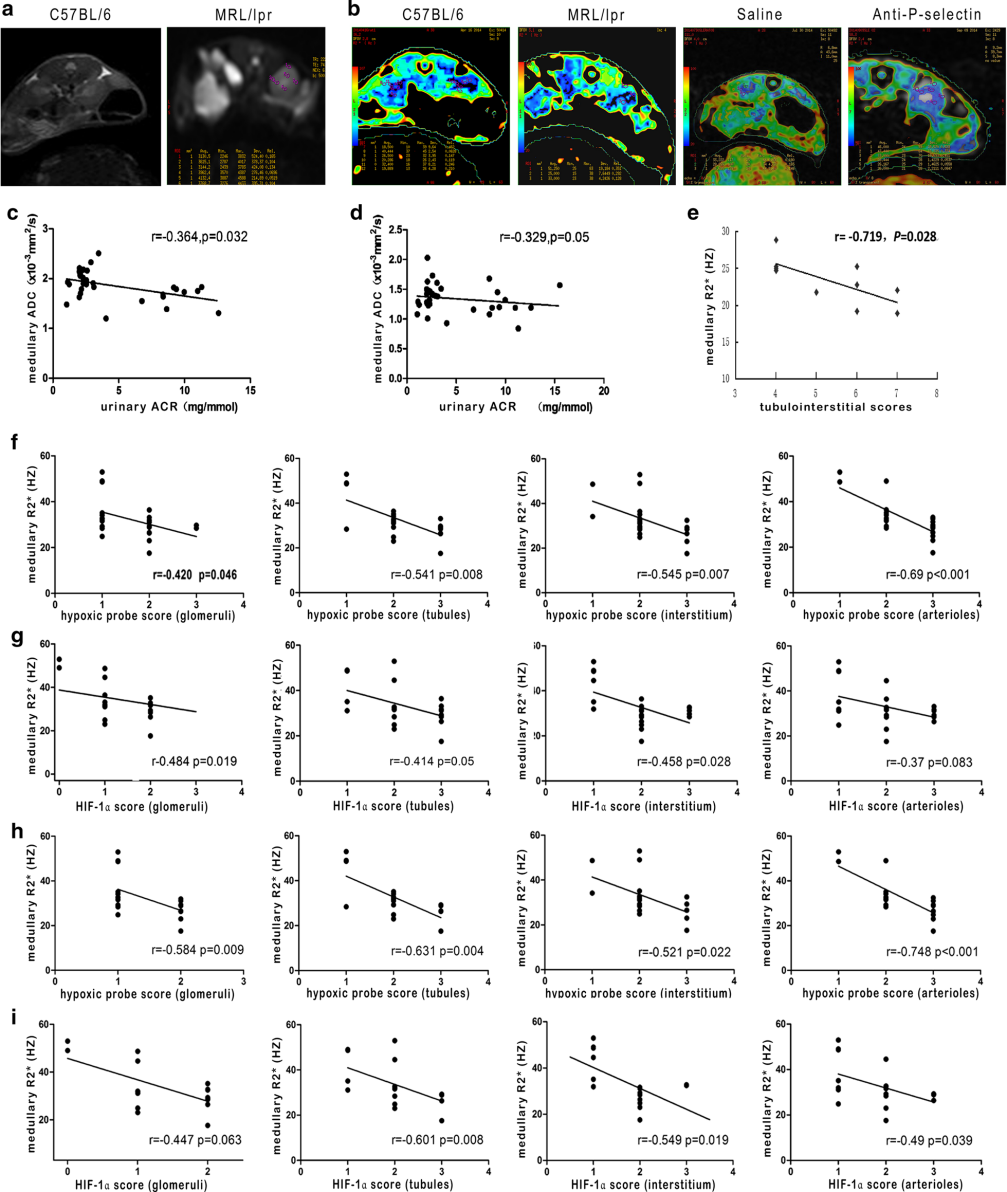
Evaluation of renal hypoxia in MRL/lpr mice through BOLD-MRI. **a** Representative T2* images of C57BL/6 and MRL/lpr mice. **b** Representative R2* images in the C57BL/6, MRL/lpr, saline, and anti-P-selectin groups. **c** Correlation between medullary ADC and urinary ACR in the C56BL/6 and MRL/lpr groups when b = 500 s/mm^2^. **d** Correlation between medullary ADC and urinary ACR in the C56BL/6 and MRL/lpr groups when b = 800 s/mm^2^. **e** Correlation between medullary R2* and tubulointerstitial scores in the C56BL/6 and MRL/lpr groups. **f** Correlation between medullary R2* and hypoxic probe score of glomeruli, tubules, interstitium, and arterioles, respectively, in the C56BL/6 and MRL/lpr groups. **g** Correlation between medullary R2* and HIF-1α score of glomeruli, tubules, interstitium, and arterioles, respectively, in the C56BL/6 and MRL/lpr groups. **h** Correlation between medullary R2* and hypoxic probe score of glomeruli, tubules, interstitium, and arterioles, respectively, in the saline and anti-P-selectin groups. **g** Correlation between the medullary R2* and HIF-1α score of glomeruli, tubules, interstitium, and arterioles, respectively, in the saline and anti-P-selectin groups

The R2* image showed that the cortex and medulla in C57BL/6 mouse kidney was clearly defined, and the color of the kidney from the cortex to the medulla was gradually altered from blue to green and then to yellow when comparing the R2* color images to the T2* gray images (Fig. 5a, b). In the LN and saline groups, because of the abnormal enlargement of the abdominal lymph nodes, the kidney was squeezed to disposition and the structure blurred between the cortex and medulla (Fig. 5a, Line 2) as compared with that in the R2* color map, in which the whole kidney was almost covered by blue (Fig. 5b, Line 2). The normal saline intervention had no obvious effect on the renal R2* map of MRL/lpr mice, while P-selectin blockade recovered the clear boundary between the renal cortex and medulla in MRL/lpr mice (Fig. 5b, Line 3 and 4). The renal cortical and medulla R2* values of MRL/lpr mice were lower than those of normal control C57BL/6 mice, and the anti-P-selectin mAb intervention induced the R2* values of renal cortex and medulla of MRL/lpr mice recovery (Table 2).

**Table 2:** Cortical and medullary R2* values of kidneys in mice

	Cortical R2* value (1/s)	Medullary R2* value (1/s)
C57BL/6	39.80 ± 4.30*	28.94 ± 0.77*
MRL/lpr	30.96 ± 4.54	23.31 ± 2.16
Norma saline treatment	28.52 ± 2.59	23.17 ± 2.96
Anti-P-selectin mAb treatment	46.37 ± 9.7**^,##^	30.16 ± 4.26*^,#^

*P < 0.05, **P < 0.01 compared with the untreated MRL/lpr group. ^**#**^*P *< 0.05, ^**##**^*P *< 0.01 compared with the same age saline group

In the C56BL/6 and MRL/lpr groups, medullary apparent diffusion coefficient (ADC) values were negatively correlated with urinary ACR when b = 500 s/mm^2^ (Fig. 5c) or 800 s/mm^2^ (Fig. 5d) but uncorrelated when b = 200 s/mm^2^. The medullary R2* value of MRL/lpr mice was negatively correlated with the tubulointerstitial lesion score (Fig. 5e) and was also significantly negatively correlated with the expression of Hypoxyprobe™-1 hypoxia probe (Fig. 5f) or HIF-1α (Fig. 5g) in glomeruli, tubules, interstitium, and arterioles. Similarly to the former two groups, in LN mice treated with saline or anti-P-selectin mAb, the mean medullary R2* values of the kidney were significantly negatively correlated with the expression of Hypoxyprobe™-1 hypoxia probe in glomeruli, tubules, interstitium, and arterioles (Fig. 5h) and were also significantly negatively correlated with the expression of HIF-1α in tubules, interstitium, and arterioles but not glomeruli (Fig. 5i).

## Discussion

Previous studies on LN have primarily focused on glomerular damage, but in recent years, tubulointerstitial lesions have been found to determine the renal prognosis of patients with LN [13]. The most important factor affecting tubulointerstitial lesions is chronic hypoxia, which leads to metabolism disorders, thereby inducing inflammatory reactions and reactive oxygen species (ROS) and causing or aggravating structural and functional damage of tubular epithelial cells and renal pathological changes [14, 15]. In the process of tubulointerstitial lesions of LN, the deposition of immune complexes in the renal tubular basement membrane and T cell–mediated immune damage may lead to endothelial cell activation, tissue ischemia and hypoxia, and massive production of ROS in the kidney. Neutrophils adhered and aggregated under the co-affection of ROS, P-selectin, and mitogen-activated protein kinase and accelerated tubulointerstitial damage [5, 16]. When renal tubular cells encounter hypoxia, fatty acid oxidation is impaired, and glycolysis becomes the main energy source. Due to these metabolic restrictions, the proximal tubular epithelial cells undergo the phenotypic transformation, including cytoskeletal rearrangement and extracellular matrix protein synthesis [17].

Previous studies have shown that MRL/lpr mice might be an appropriate model for studying LN, because they have a good consistency with the process of human LN. In our study, they showed elevated serum anti–ds-DNA antibody levels and proteinuria compared with C57BL/6 mice but no difference in serum complement C3 and creatinine levels (data not provided). The abnormality of the immune index in the MRL/lpr mouse model is mainly characterized by high-titer anti-nuclear antibody and anti-DNA antibody, but the decrease of complement is not obvious [13]. In fact, we raised MRL/lpr mice to 20 weeks of age, and serum creatinine levels remained unchanged from 12 weeks of age. Passwell et al. [18] showed that slight serum creatinine elevation could be detected in the MRL/lpr strain mice until 22 weeks of age, although the average life span was only 5 months in females and 5.5 months in males [19]. The MRL/lpr mice developed a mild to moderate mesangial hypercellularity 12 weeks of age, which evolved into obvious mesangioproliferation with tubulointerstitial lesion at 16 weeks old [20], Moreover, our findings confirmed the renoprotective role of the anti-P-selectin mAb intervention at this stage.

Cell adhesion molecules are cell-membrane proteins that maintain cell–cell and cell-substrate adhesion and transduce intracellular signaling in some cases. Adhesion molecules are generally divided into five groups: integrins, selectins, cadherins, members of the immunoglobulin superfamily (IgSF) including nectins and others such as mucins [21]. In the kidney, integrins and cadherins are essential for maintaining the epithelial polarity and barrier integrity, while some members of the IgSF and the selectins (ICAM-1, VCAM-1, E-selectin and P-selectin) are essential mediators of both immune and inflammatory responses. P-selectin is a major member of the cell adhesion molecule family, and its role in various glomerular diseases has also received extensive attention [22–24]. P-selctin participates in trafficking of cells of the innate immune system, T lymphocytes and platelets and is involved in constitutive lymphocyte homing and in chronic and acute inflammation processes, including bacterial infections, glomerulonephritis and SLE [25]. Its expression is significantly upregulated in plasma and renal tissues of patients with LN. P-selectin and its mediated infiltration of dendritic cells and accumulation in renal interstitium initiated a tubulointerstitial inflammatory immunoreaction and histopathological damage in the early stage of LN [26, 27]. However, He et al. [28] found that P-selectin knockout in MRL/lpr mice was not protective but rather accelerated glomerulonephritis with upregulated expression of the chemokine CCL2 in both kidney tissue and in purified renal endothelial cells. In this research, we found that the expression of P-selectin in the MRL/lpr mouse kidney tissue, especially the tubulointerstitium, increased as compared with that in C57BL/6 mice, and the expression was more significant as the degree of renal lesions increased. P-selectin blockade decreased the urinary protein levels in MRL/lpr mice, which was more significant in mice who underwent a 4-week intervention that commenced from 12 weeks of age as compared with 16 weeks of age. In addition, we noted improved renal tubular necrosis and inflammatory cell massive accumulation in the interstitium and pericapillary. We also found that anti-P-selectin mAb had no direct effect on anti–ds-DNA antibody or complement C3 in LN mice, suggesting that the immune mechanisms of P-selectin on LN progression involve infiltration and differentiation of T cells and neutrophils rather than B-cell antibody secretion or the complement activation system.

Under normal conditions, the oxygen partial pressure of the renal cortex (about 50 mmHg) is significantly higher than that in the medulla (10–20 mmHg), causing the medulla to become sensitive to hypoxia and severe tubulointerstitial lesions during stress [29, 30]. The interstitial blood supply derives from postglomerular blood vessels; thus, glomerular hypertension and sclerosis can cause interstitial ischemia, while the interstitial oxidative stress due to inflammation aggravates hypoxia [1]. We employed the Hypoxyprobe™-1 hypoxia probe and hypoxia marker HIF-1α to evaluate renal hypoxia in LN mice and found that their distribution were consistent with a more positive expression in the medulla than that in the cortex, suggesting that there is diffuse hypoxia in the medulla of LN model mice. Hypoxyprobe™-1 is a hypoxia marker of cells or tissues, and HIF-1α reflects the ratio of local oxygen supplement to demand. The HIF-1α pathway is activated when the demand for oxygen is greater than oxygen supplement [31, 32]. In severe tubulointerstitial lesions, the expression of the hypoxia probe and HIF-1α was upregulated in renal tissues, suggesting that renal hypoxia plays an important role in the development of LN tubulointerstitial lesions. However, P-selectin blockade recovered the expression of hypoxia probe Hypoxyprobe™-1 and HIF-1α, indicating that LN renal hypoxia was significantly reduced after inhibition of P-selectin. Changes in the expression of NADPH oxidase subunits gp91phox and p22phox suggested that oxidative stress was also involved in this process. Based on these findings, it is speculated that in the early stage of LN, P-selectin is upregulated in renal tissue, mediating rolling of leukocytes and adhesion to vascular endothelial cells. On one hand, leukocytes cross the blood vessels to invade the interstitial space, causing interstitial inflammation and injury to vascular endothelial cells. On the other hand, the density of capillaries in the renal medulla decreases with endothelial cell injury, so that their supplied oxygen and nutrients are greatly reduced, causing hypoxia and tubulointerstitial lesions in the kidney. Hypoxia stimulates endothelial cells to express P-selectin, forming a vicious circle. This suggests that the improvement of tubulointerstitial hypoxia may be the mechanism by which P-selectin monoclonal antibody relieves renal tubulointerstitial fibrosis. Blocking early P-selectin-mediated leukocyte adhesion may have therapeutic implications in improving the prognosis of LN. At present, the human monoclonal antibody against P-selectin, inclacumab, has passed phase II clinical trials in coronary interventions [33, 34], and our findings reveal its potential for the treatment of LN.

In this study, BOLD-MRI showed that the R2* value of the mouse kidney medulla was negatively correlated with the urinary protein level, suggesting that when there is a large amount of proteinuria during glomerular lesions, it is often accompanied by insufficient renal blood perfusion, which can lead to renal tissue, the medulla in particular, ischemia and hypoxia, causing a decrease in the medulla R2* value. In the LN model mice, the renal medulla R2* value was lower than the cortical R2* value, which was consistent with the distribution of the hypoxia probe and HIF-1α in the kidney, indicating that BOLD-MRI can noninvasively evaluate the intrarenal oxygenation level to assess tubulointerstitial damage and prognosis. The blurred boundary between the renal cortex and medulla was recovered in MRL/lpr mice after P-selectin blockade, and the R2* color map reflected the improvement of the hypoxic state in the kidney, after which the metabolism of the kidney cells improved and the oxygen demand increased, so that the R2* value of the cortex and medulla increased. In addition, there was no significant correlation between the cortex medulla ADC or R2* values and serum anti–ds-DNA antibody or complement C3 levels, suggesting that immune factors have no direct effect on renal ADC values or R2* values.

## Conclusions

This study showed that P-selectin is upregulated in LN kidney tissue and mediates endothelial-leukocyte adhesion, which in turn causes vascular endothelial injury, intrarenal hypoxia, and inflammatory cell infiltration and plays an important role in LN tubulointerstitial lesions. Early intervention of LN with anti-P-selectin mAb can significantly improve the hypoxic state of the kidney and reduce the severity of tubulointerstitial lesions, providing a new idea for the diagnosis and treatment of LN clinically. Functional MR DWI and BOLD-MRI techniques are noninvasive and can dynamically evaluate the changes in renal lesions and intrarenal oxygenation levels before and after treatment in LN, which provides potential clinical value.

## Data Availability

All data generated or analyzed during this study are included in this article.

## Abbreviations

LN: Lupus nephritis
DWI: Diffusion-weighted imaging
BOLD: Blood oxygen level dependent
MR: Magnetic resonance
SE-EPI: Spin echo–echo planar imaging
mGRE: Multiple gradient-recalled-echo
TR: Repetition time
TE: Echo time
ADC: Apparent diffusion coefficient
ROI: Region of interest
AI: Activity index
CI: Chronicity index
CR: Complete remission
NR: No response
ESRD: End-stage renal disease
